# Evaluation of the linkage-disequilibrium method for the estimation of effective population size when generations overlap: an empirical case

**DOI:** 10.1186/s12864-015-2167-z

**Published:** 2015-11-11

**Authors:** María Saura, Albert Tenesa, John A. Woolliams, Almudena Fernández, Beatriz Villanueva

**Affiliations:** Departamento de Mejora Genética Animal, INIA, Carretera de la Coruña km 7.5, 28040 Madrid, Spain; The Roslin Institute and R(D)SVS, University of Edinburgh, EH25 9RG, Midlothian, UK

**Keywords:** Effective population size, Genome-wide information, Iberian pig, Linkage disequilibrium, Overlapping generations, Pedigree

## Abstract

**Background:**

Within the genetic methods for estimating effective population size (*N*_*e*_), the method based on linkage disequilibrium (LD) has advantages over other methods, although its accuracy when applied to populations with overlapping generations is a matter of controversy. It is also unclear the best way to account for mutation and sample size when this method is implemented. Here we have addressed the applicability of this method using genome-wide information when generations overlap by profiting from having available a complete and accurate pedigree from an experimental population of Iberian pigs. Precise pedigree-based estimates of *N*_*e*_ were considered as a baseline against which to compare LD-based estimates.

**Methods:**

We assumed six different statistical models that varied in the adjustments made for mutation and sample size. The approach allowed us to determine the most suitable statistical model of adjustment when the LD method is used for species with overlapping generations. A novel approach used here was to treat different generations as replicates of the same population in order to assess the error of the LD-based *N*_*e*_ estimates.

**Results:**

LD-based *N*_*e*_ estimates obtained by estimating the mutation parameter from the data and by correcting sample size using the 1/2*n* term were the closest to pedigree-based estimates. The *N*_*e*_ at the time of the foundation of the herd (26 generations ago) was 20.8 ± 3.7 (average and SD across replicates), while the pedigree-based estimate was 21. From that time on, this trend was in good agreement with that followed by pedigree-based *N*_*e*_.

**Conclusions:**

Our results showed that when using genome-wide information, the LD method is accurate and broadly applicable to small populations even when generations overlap. This supports the use of the method for estimating *N*_*e*_ when pedigree information is unavailable in order to effectively monitor and manage populations and to early detect population declines. To our knowledge this is the first study using replicates of empirical data to evaluate the applicability of the LD method by comparing results with accurate pedigree-based estimates.

**Electronic supplementary material:**

The online version of this article (doi:10.1186/s12864-015-2167-z) contains supplementary material, which is available to authorized users.

## Background

The effective size of a population (*N*_*e*_) is the size of an idealized population that would show the same amount of genetic drift or the same inbreeding rate than the population under consideration [1]. It is an important parameter in population and quantitative genetics and in evolutionary and conservation biology because it determines the rate at which genetic variation is lost.

Traditionally, *N*_*e*_ has been estimated from demographic or pedigree data [2], but when this information is unavailable, *N*_*e*_ can be estimated from genetic data [3]. Until recently, the most widely used genetic method for estimating contemporary *N*_*e*_ has been the temporal method [4, 5] which is based on the temporal changes in allele frequencies for samples of the same population collected at (at least) two different points in time. However, with the advent of high throughput genotyping techniques, the one-sample method based on linkage disequilibrium (LD) measures [6] has attracted increasing attention in recent years [7–13].

One of the main advantages of the LD-based method over the temporal method is that it uses much more information and therefore leads to estimates of higher accuracy [7, 13, 14]. Also, the strength of LD at different genetic distances between loci can be used to infer *N*_*e*_ at any point in time from a single sample [13], as LD between a pair of markers is determined by the product of *N*_*e*_, recombination frequency and number of generations since mutation [15]. On the contrary, the temporal method only gives estimates of *N*_*e*_ over the period between sample collections.

An important assumption of the genetic methods proposed for estimating *N*_*e*_ that is often violated is that generations are discrete. Different corrections have been developed to account for overlapping generations when using the temporal method [16, 17], but the applicability of the LD method in this context remains unclear [14]. It is also unclear the optimal way to account for different factors that affect the relationship between LD and *N*_*e*_ such as mutation and sample size [6] under a scenario of overlapping generations.

Under this context, a valuable dataset for investigating the applicability of the LD method would be that coming from a population with overlapping generations where accurate *N*_*e*_ estimates across generations can be obtained (e.g., from pedigree data). These accurate estimates could then be used as a baseline against which to compare LD-based estimates. This is the case of the present study, where complete and accurate pedigree records (from where accurate pedigree-based *N*_*e*_ estimates can be obtained) for an experimental herd of Iberian pigs with overlapping generations founded in 1944 are available. Our aim was to obtain estimates of *N*_*e*_ with the LD method using genome-wide data obtained with the Illumina PorcineSNP60 BeadChip. By comparing estimates for *N*_*e*_ assuming different models that varied in the way in which mutation and sample size were accounted for with accurate pedigree-based *N*_*e*_ estimates it was possible to determine the most appropriate model. Another novelty implemented in this study was to treat different generations as replicates of the same population, in order to assess the error of the estimates. To our knowledge, this is the first study that evaluates the applicability of the LD method in a population with overlapping generations using temporal replicates of empirical data and accurate estimates of pedigree-based *N*_*e*_ as a benchmark to decide the optimal model for obtaining LD-based estimates.

## Methods

### Ethical statement

The current study was carried out under a Project License from the INIA Scientific Ethic Committee. Animal manipulations were performed according to the Spanish Policy for Animal Protection RD1201/05, which meets the European Union Directive 86/609 about the protection of animals used in experimentation. We hereby confirm that the INIA Scientific Ethic Committee, which is the named IACUC for the INIA, specifically approved this study.

### Samples and SNP genotypes

Data used in this study originated from pigs from the Guadyerbas strain that represents one of the original strains of Iberian pigs [18]. It has been kept isolated in an experimental closed herd established from 24 founders (4 males and 20 females) in 1944. Management of the herd has implied avoidance of matings between relatives. Very accurate and complete pedigree data have been recorded since the time of the foundation of the herd until 2011 (26 generations). The complete genealogy includes 1,178 animals.

Genotypic data from the Illumina Porcine SNP60 BeadChip v1 were available for 227 Guadyerbas animals born between 1992 and 2011 in the herd. This implies that genotypes were available for all the animals belonging to the last 6 generations (generations 21 to 26). The Illumina Porcine SNP60 BeadChip v1 contains 62,163 probes that are distributed along 18 autosomal and two sex chromosomes, according to the latest version of the porcine map (Sscrofa 10.2). DNA samples, extracted from blood samples were hybridized with the chip and images were scanned by an external service (Universidad Autónoma de Barcelona, Spain). Genotype calls were obtained with the Genotyping Module of the GenomeStudio Data Analysis software (Illumina Inc.). Quality control criteria used were those described in Saura et al. [19]. In brief, a total of 468 samples (227 Guadyerbas and 241 samples from other strains of the Iberian breed) were used to check the quality of genotyping data. SNPs that did not satisfy the following quality control criteria were removed: Call Frequency < 0.99, GenTrainScore < 0.70, AB R Mean < 0.35, MAF = 0 and number of inconsistencies with the genealogy > 9. Unmapped SNPs and SNPs mapped to sex chromosomes were also excluded. After filtering SNPs, the data were reanalysed and samples with a Call Rate < 0.96 and with a large number of inconsistencies with the genealogy were removed. The final number of autosomal SNPs and Guadyerbas samples available for the analysis were 219 and 35,519, respectively. The number of SNPs segregating was 19,145. Further information about estimates of genetic variability in this herd can be found in Fernández et al. [20] and Saura et al. [19]. The number of genotyped animals per generation was 35, 52, 42, 27, 15 and 48 for generations 21, 22, 23, 24 25 and 26, respectively.

### Estimation of *N*_*e*_ across time from pedigree data

Pedigree-based estimates of *N*_*e*_ per generation were obtained from the rate of inbreeding per generation (Δ*F*) as *N*_*e*_ = (2*ΔF*)^− 1^ and Δ*F* was estimated from individual long-term contributions to the last generation [21–23]; i.e., $\varDelta F=\left({\displaystyle {\sum}_{i=1}^{N_t}{c}_i^2}\right)/4$, where *c*_*i*_^2^ is the squared long-term contributions of individual *i* and *N*_*t*_ is the number of individuals that form generation *t*. The long-term genetic contribution of an ancestor [22, 24, 25] is the ultimate proportional contribution of the ancestor to generations in the distant future [26]. Thus, the effective population size at generation *t* was obtained as ${N}_{e(t)}=2/\left({\displaystyle {\sum}_{i=1}^{N_t}{c}_i^2}\right)$. Long-term contributions were computed using a Fortran software provided by DM Howard (The Roslin Institute, personal communication). An example of the calculation of long-term contributions is provided in the Additional file 1.

The generation interval (*L*) is defined as the turnover time of genes which equal to the time in which genetic contributions sum to unity; i.e., the genetic contribution summed over all ancestors entering the population over a time period of *L* years equals unity: (∑*c*_*i*_) = 1 [26]. Here, the sum of long-term contributions is taken over those born over the time unit (years).

### LD estimation

Estimates of *N*_*e*_ were obtained on a per generation basis. Given that we are dealing with overlapping generations, a given generation included a number of consecutive cohorts that was roughly equal to *L*. According to this and given that genotypes for the last six generations were available, we built six replicates of the same population and each replicate corresponded to one generation. This design of splitting the data into replicates has never been considered before. It allowed us to evaluate the error of the *N*_*e*_ estimates.

For each replicate (i.e., for each generation), autosome pairwise LD was computed as the squared correlation between pairs of SNPs (*r*^*2*^) [15] using the formula

$$ {r}^2=\frac{D^2}{p_A{p}_a{p}_B{p}_b}, $$

where *D* = *p*_*AB*_ − *p*_*A*_*p*_*B*_, *p*_*AB*_ is the frequency of the genotype *AB* (coupling phase) and *p*_*A*_, *p*_*a*_, *p*_*B*_ and *p*_*b*_ are the frequencies of alleles *A*, *a*, *B* and *b*, respectively. Values for *r*^*2*^ were obtained using the software provided by R Pong-Wong (The Roslin Institute, personal communication) which implements an EM algorithm [27] to estimate haplotype frequencies. To enable a clear presentation of results showing LD in relation to physical distance between markers, SNP pairs were divided into three distance classes: (*i*) 0 to 2 Mb, (*ii*) 2 to 5 Mb and (*iii*) 5 to 50 Mb. Distance bins of 0.05, 0.20 and 5.00 Mb were used for classes (*i*), (*ii*) and (*iii*), respectively, and average *r*^*2*^ values for each bin were plotted against physical distance. Also, in order to determine the background LD (i.e., the LD expected by chance), *r*^*2*^ was calculated for a random selection of non-syntenic SNPs that included 30 SNPs per autosome, following Khatkar et al. [28]. Thus, 137,700 pairwise independent correlations were computed.

### Estimation of *N*_*e*_ across time from LD

The well known relationship between LD and *N*_*e*_ was derived by Sved [29] and it was based on the work of Hill and Robertson [15]. However, Sved’s equation did account neither mutation nor the effect of sample size. The consequence of ignoring mutation is that even with physical linkage, the expected association between loci is incomplete and leads to an underestimation of *r*^*2*^. This effect increases as historical time increases. The consequence of ignoring sample size is that the magnitude of *r*^*2*^ can be overestimated in samples of small size as a consequence of spurious associations. This effect increases as historical time decreases [11]. Taking into account both mutation and sample size leads to a more general expression:

$$ E\left[{r}^2\right]={\left(\alpha +4{N}_ec\right)}^{-1}+1/N $$

where *α* is a parameter related to mutation, *c* is the recombination rate and the term 1/*N* is an adjustment for sample size [30]. Note that 4*N*_*e*_ corresponds to the slope and *α* to the intercept of the regression equation. Parameter *α* can be set to a fixed value (1 in the absence of mutation or 2 when mutation is considered) [31, 32] but can also be estimated from the data. When haplotypes are unknown (i.e. coupling and repulsion double heterozygotes cannot be distinguished), *N* equals the number of diploid individuals sampled (*n*) [30], whereas when haplotypes are known without error *N* equals 2*n* [33]. Here, the effect of correcting for mutation and sample size was investigated by comparing results from six different models that varied in *α* and 1/*N* as shown in Table 1.

**Table 1:** Description of the statistical models assumed to estimate *N*_*e*_ based on LD

Model	α	Sample size correction
a	Estimated	None
b	Estimated	1/2*n*
c	Estimated	1/*n*
d	Fixed to 2	None
e	Fixed to 2	1/2*n*
f	Fixed to 2	1/*n*

Six different models differing in mutation and sample size adjustments are indicated

*n* number of individuals

For predicting *N*_*e*_ at any generation in the past, we have to consider *r*^*2*^ between SNP pairs at a specific linkage distance in Morgans (*d*). This distance yields a prediction of the time since the gametes are expected to have coalesced 1/(2*c*) generations ago [34, 35]. Thus,

$$ {N}_{e(t)}={(4d)}^{-1}\left[{\left({r}_d^2-{N}^{-1}\right)}^{-1}-\alpha \right] $$

where *N*_*e(t)*_ is the effective population size *t* generations ago and *r*_*d*_^2^ is the mean value of *r*^*2*^ for markers *d* Morgans apart. Based on this equation a non-linear least squares approach was implemented to statistically model the observed *r*^*2*^. With the aim to avoid dependence between LD and linkage distance estimated from the same data, we used estimates of recombination rates for each chromosome from a different study in the same species. Specifically, we used estimates from Tourtereau et al. [36] who described a high density recombination map for the pig using the Porcine SNP60 BeadChip. They built this map using information from four independent pedigrees coming from F2 crosses involving five breeds (Berkshire, Duroc, Meishan, Yorkshire and Landrace). Therefore, for each SNP pair in LD separated by a particular physical distance (Mb), an equivalent linkage distance (Morgans) was calculated as the product of the recombination rate for a particular chromosome obtained from Tourtereau et al. [36] and the physical distance.

We investigated the evolution of *N*_*e*_ across the period between 1944 (year of the foundation of the herd) and 2011 (the last year for which data were available hereinafter referred to as ‘the present’), a period comprising 26 generations. To estimate *N*_*e*_ at the time of the foundation of the herd (i.e. 26 generations ago) from the relationship between *t* and *d*, we had to consider SNP pairs at a linkage distance of approximately 0.019 Morgans which in this population corresponded to 2.5 Mb. In the same way, to calculate the *N*_*e*_ at the present, the corresponding physical distance between the SNP pairs corresponded approximately to 66 Mb. However, non-syntenic levels of LD might be taken into account, as they are not a function of the distance. Otherwise *N*_*e*_ estimates will be biased. In particular, the maximum distance between SNP pairs that can provide informative estimates of *N*_*e*_ needs to be lower than the distance determining non-syntenic levels of LD. In addition, to ensure that sufficient SNP pairwise comparisons are available to obtain estimates of *r*^*2*^ with acceptable precision, we used a maximum distance between SNPs of 15 Mb (from which 180 LD measures were obtained), which translated into 5 generations in the past.

We segmented the interval between 2.5 and 15.0 Mb into 63 distance categories (i.e. from 2.5 Mb and increasing by 0.2 Mb consecutive categories up to 15.0 Mb) and performed an equivalent number of regressions to estimate *N*_*e(t)*_ at different times. We applied this procedure on each of the six scenarios differing in mutation and sample size adjustments and on each of the six replicates.

## Results

### Estimation of *N*_*e*_ across time from pedigree data

**Fig. 1:**
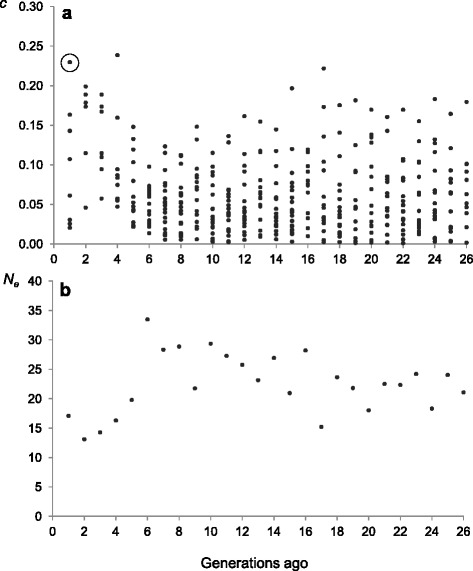
Individual long-term contributions (*c*) and estimates of effective population size (*N*_*e*_) based on those contributions. Long-term contributions for each individual to the last generation obtained from pedigree information (**a**) and estimates of *N*_*e*_ obtained from individual contributions plotted against generations in the past (**b**). Each dot in (**a**) represents the contribution of one individual born ‘x’ generations ago to the last generation. For instance, the circled dot represents the contribution of an individual born one generation ago (i.e., generation 25) to the last generation (i.e., generation 26)

Figure 1 shows estimates of long-term contributions and *N*_*e*_ obtained from pedigree data. The estimated *N*_*e*_ at the time where the herd was founded 26 generations ago was 21 individuals. After that, the value for *N*_*e*_ slightly increased across generations up to six generations ago, when a decrease of 40 % in *N*_*e*_ was observed. The average generation interval estimated from long-term contributions was approximately three years.

These estimates were used as the reference estimates with which to compare the corresponding *N*_*e(t)*_ obtained from LD measures under the different models assumed. In this way, the molecular estimates that better fit the pedigree estimates will determine the best model of adjustment.

### LD estimates

**Fig. 2:**
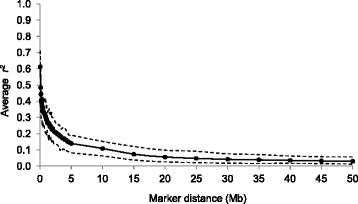
Average linkage disequilibrium plotted against distance between SNPs. Average linkage disequilibrium (solid line) measured as *r* ^2^ and the 5th and 95th percentiles (dashed lines) plotted against the average of the distance bin range (Mb). To enable a clear presentation of results, distances between SNP pairs were divided into three distance ranges: (i) 0 to 2 Mb, (ii) 2 to 5 Mb and (iii) 5 to 50 Mb. Distance bins of 0.05, 0.20 and 5.00 Mb were used for classes (i), (ii) and (iii), respectively, and average *r* ^*2*^ values pooled over autosomes for each bin were plotted against physical distance

The average *r*^*2*^ between adjacent SNP was 0.53 (±0.42) and the mean distance between adjacent SNP was 0.068 Kb. Figure 2 represents the decline in *r*^*2*^ between syntenic SNP pairs (averaged across all autosomes and replicates) with increasing distance. The average *r*^*2*^ for SNPs at distances between 0.00 and 0.05 Mb was 0.61. The most rapid decline in *r*^*2*^ was observed at marker distances lower than 0.90 Mb where *r*^*2*^ decreased by more than half. Table 2 summarizes the average *r*^*2*^ for each autosome for different categories of distances between markers up to 50.00 Mb. The average *r*^*2*^ was reduced to non-syntenic levels (0.017 ± 0.029) at distances greater than 50.00 Mb.

**Table 2:** Levels of LD for different distance categories between SNP pairs for the different autosomes

Chr	N_SNP_	0.5 Mb	1.0 Mb	5.0 Mb	10.0 Mb	50.0 Mb
SSC1	3953	0.24	0.21	0.13	0.10	0.06
SSC2	2462	0.42	0.37	0.24	0.18	0.09
SSC3	1868	0.46	0.41	0.28	0.22	0.11
SSC4	2337	0.49	0.44	0.28	0.20	0.07
SSC5	1567	0.40	0.36	0.22	0.16	0.07
SSC6	2352	0.48	0.42	0.28	0.21	0.08
SSC7	2295	0.38	0.33	0.21	0.16	0.07
SSC8	2064	0.50	0.45	0.28	0.21	0.11
SSC9	2308	0.41	0.37	0.24	0.18	0.08
SSC10	1217	0.32	0.28	0.17	0.12	0.05
SSC11	1410	0.37	0.31	0.19	0.14	0.06
SSC12	1022	0.38	0.33	0.18	0.13	0.06
SSC13	2802	0.52	0.47	0.31	0.25	0.16
SSC14	2530	0.47	0.42	0.28	0.21	0.10
SSC15	2099	0.44	0.40	0.26	0.20	0.10
SSC16	1134	0.42	0.36	0.22	0.18	0.08
SSC17	1054	0.47	0.41	0.26	0.20	0.09
SSC18	1045	0.39	0.34	0.20	0.14	0.06
Average		0.42	0.37	0.24	0.18	0.08

*Chr* chromosome

*N*_*SNP*_ number of SNPs

### Estimation of *N*_*e*_ across time from LD

**Fig. 3:**
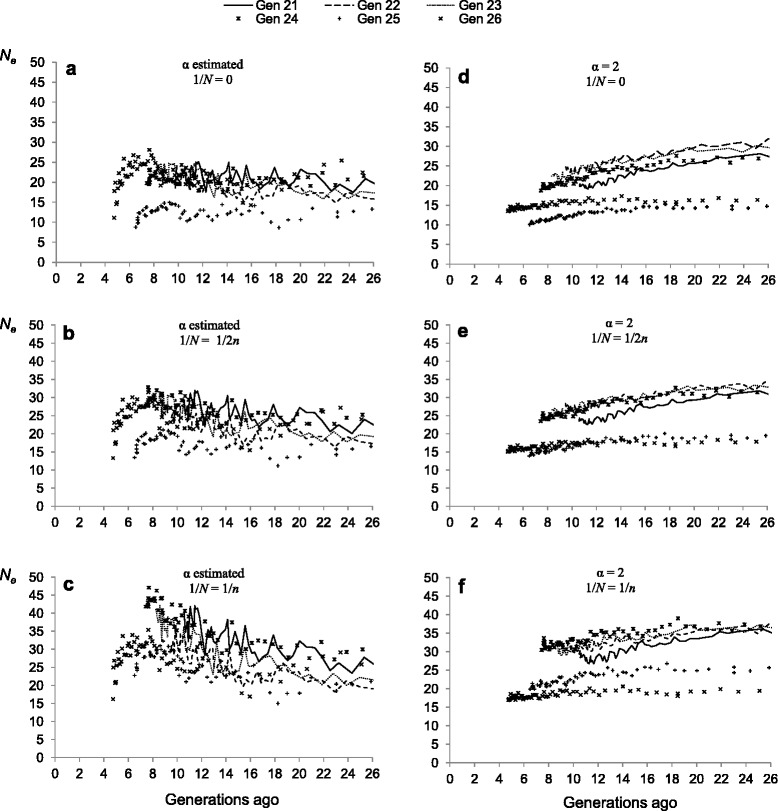
Estimates of effective population size (*N*_*e*_) based on linkage disequilibrium plotted against generations in the past. Estimates of *N*_*e*_ obtained from linkage disequilibrium measures for the six temporal replicates (generations, Gen) estimating (**a**, **b** and **c**) or fixing α to 2 (**d**, **e** and **f**) and ignoring (**a** and **d**) or accounting (**b**, **c**, **e** and **f**) for the sampling effect. The term 1/*N* is the adjustment for sample size

Figure 3 shows estimates of *N*_*e(t)*_ based on LD for the time period elapsed between the foundation of the herd and the present, for the six models assumed and for the six temporal replicates. Each point in the graphs corresponds to the result of a non-linear regression for a specific physical distance category, as previously explained.

**Fig. 4:**
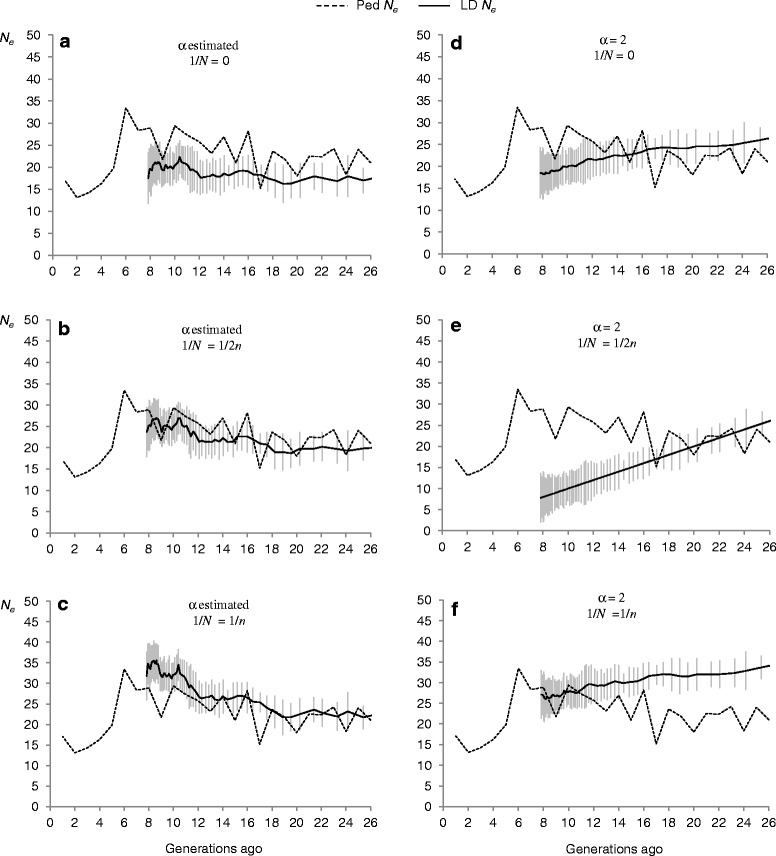
Comparison of estimates of effective population size (*N*_*e*_) based on linkage disequilibrium with those based on pedigree data, averaged across replicates. Average LD-based *N*_*e*_ estimates across replicates (solid lines; bars represent 1 SD) compared to pedigree-based estimates (dashed lines). Estimates based on LD were obtained estimating (**a**, **b** and **c**) or fixing α to 2 (**d**, **e** and **f**) and ignoring (**a** and **d**) or accounting (**b**, **c**, **e** and **f**) for the sampling effect. The term 1/*N* is the adjustment for sample size

In general, average values of *N*_*e*_ across replicates at the time of the foundation of the herd were lower when *α* was estimated than when it was fixed. Standard deviations of the *N*_*e*_ estimate across replicates were higher in models where *α* was fixed (17.5 ± 3.5, 20 ± 3.6, 22.5 ± 3.5 for scenarios a, b, c, respectively, and 26.5 ± 8.5, 26.5 ± 8.5 and 34.5 ± 9.5 for scenarios d, e, f, respectively). All scenarios analyzed provided estimates significantly different from zero for both *α* (scenarios a, b and c) and *N*_*e*_. With the exception of model c (Fig. 3c), models where *α* was estimated from the data led to *N*_*e*_ estimates that slightly increased from the time of the foundation of the herd to approximately six generations ago, when a decline occurred. Models where *α* was fixed to 2 led to *N*_*e*_ estimates that decreased progressively across generations (Fig. 3d, e, f). The molecular *N*_*e(t)*_ estimates closest to pedigree-based estimates were those obtained under the model where (*i*) the parameter *α* was estimated; and (*ii*) a correction of 1/2*n* for sample size was imposed (i.e., model b, Figs. 1 and 4b).

## Discussion

In this study we have evaluated the applicability of the LD method when using genome-wide information for estimating *N*_*e*_ in an experimental population of Iberian pigs where generations overlap. We took advantage of having a complete and accurate pedigree dataset available, a situation that is very rare in practice. This allowed us to obtain precise pedigree-based estimates of *N*_*e*_ that were considered as a baseline against which to compare LD-based *N*_*e*_ estimates. The approach allowed us to determine the most suitable statistical model of adjustment when the LD method is used for species with overlapping generations. This is in a context of conserved populations of small *N*_*e*_ for which genetic methods perform better because the signal (drift in allele frequencies or LD) is the largest [13]. The LD-based *N*_*e*_ estimates closest to pedigree-based estimates were those obtained under model b, that adjusted the values of *r*^*2*^ for sample size using the 1/2*n* correction and estimated *α* from the data (SD were the lowest when estimating *α* instead of fixing it). The novel use of contiguous generations as temporal replicates made also possible to determine the error of the estimates and confirmed the applicability of the method. The study represents thus an advance towards the use of the LD method to estimate *N*_*e*_ in species where generations overlap.

We have investigated for the first time the effect of different sample size corrections under the new context of having available genome-wide information and efficient analytical methods to predict *r*^*2*^ from unphased data. In theory, under a two-locus model, the 1/2*n* correction should only be applied when haplotypes are known without error; otherwise the correction to be used is 1/*n* [11]. In the particular case of the Guadyerbas strain, the inbreeding rate is high [18, 19]. In fact, both the amount and the extension of LD were much higher than those observed in other pig breeds [37–39]. A priori, the fact that high levels of inbreeding have been detected in this population would imply that the number of double heterozygotes (the only context where phases cannot be distinguished) is low. Thus, the distinction of gametic phases might be relatively straightforward when using accurate algorithms such as the EM algorithm. Also, the 1/*n* correction would probably be too strong under a scenario of small population size. In summary, model b seems to be the most appropriate model to apply in small populations with overlapping generations when high dense SNP data are used.

Another important issue to consider when applying the LD method is the coalescence time. Coalescence theory predicts that the expected time for coalescence of a sample of gametes is < 4*N*_*e*_ generations. Therefore, estimates of *N*_*e*_ more than 4*N*_*e*_ generations in the past may be questionable. Although this corresponds to 96 generations in this study, our interest was to go back to the time of the foundation of the herd (i.e. 26 generations ago) as this is the period of time for which pedigree-based estimates can be obtained and compared with LD-based estimates. Molecular estimates of *N*_*e(t)*_ under the more suitable model (*α* estimated and 1/2*n* correction used) showed that *N*_*e*_ slightly increased for 20 generations from the time of the foundation of the herd. After 20 generations *N*_*e*_ suffered a strong reduction due to a well-documented disease outbreak experienced in the herd. This result was in agreement with the pedigree-based *N*_*e(t)*_ and validates the accuracy of the method to predict population decline for populations of small *N*_*e*_, as suggested in previous studies [13, 40].

Previous studies have questioned the precision of *N*_*e*_ at recent generations [6, 11]. This precision can be influenced by (*i*) the effect of non-syntenic LD, that conditions the maximum distance between SNP pairs to compute informative *r*^*2*^ measures, and by (*ii*) the availability of sufficient SNP pair comparisons when the distance between SNPs is very high. The basal levels of non-syntenic LD (i.e. the LD expected by chance) were higher (0.017 ± 0.029) than those reported for other livestock species such as cattle (0.0032 ± 8 × 10^−7^, [28]) and horses (0.0018 ± 2.49 × 10^−3^, [10]), and for other pig breeds (no LD was found between non-syntenic SNPs, [37]). Our results of non-syntenic levels of LD were equivalent to LD levels between syntenic SNPs separated > 50.00 Mb, which had not impact on our calculations of recent *N*_*e*_. However, computation of *N*_*e*_ for the last five generations would not be adequate because there are not enough SNP pairwise correlations to ensure a high accuracy.

Specific corrections for considering overlapping generations have been suggested for different demographic and genetic methods [2, 16, 17] but not for the LD method. However, simulation studies have shown that levels of LD in samples including a number of consecutive cohorts equal to the generation length should provide accurate estimates of per-generation *N*_*e*_ for populations with overlapping generations [13, 37, 41]. Our results validate this argument.

## Conclusions

Early detection of population decline is a crucial issue to prevent extinctions by implementing management actions (monitoring, transplanting, habitat restoration, disease control, etc.) that reduce extinction risks. The maintenance of large *N*_*e*_ and the associated genetic variation is also important in terms of evolution, because loss of genetic variation affects the adaptation capability of a population. This highlights the importance of using accurate genetic estimators of *N*_*e*_ in populations where pedigree and/or demographic data are not available. Our results showed that, when using genome-wide information currently available, the LD method is accurate and broadly applicable to populations with small *N*_*e*_ even for species with overlapping generations.

## Additional file

Additional file 1:
**Example of calculation of long-term contributions.** (DOCX 14 kb)
