# Supplementary material for: Evaluation of the linkage-disequilibrium method for the estimation of effective population size when generations overlap: an empirical case
Source: BMC Genomics. 2015 Nov 11;16:922. doi: 10.1186/s12864-015-2167-z (PMC4642667; doi:10.1186/s12864-015-2167-z)
Supplement: Additional file 1: — Example of calculation of long-term contributions. (DOCX 14 kb) [file 12864_2015_2167_MOESM1_ESM.docx]

**Example of calculation of long-term contributions**

Contributions of an individual ancestor to its descendants are calculated by the following algorithm: (i) the contribution of an ancestor to itself is 1; (ii) the contribution of an ancestor to a descendant is the average of the contributions of the ancestor to each of its parents. Since contributions are concerned with inheritance of the Mendelian sampling term of an ancestor, an ancestor makes no contribution to those individuals that are not directly descended from it.

The following 4 generation pedigree is shown in standard format of individual, sire and dam, with 00 denoting unknown parent.

| 01 | 00 | 00 |  | 05 | 01 | 03 |  | 09 | 05 | 07 |  | 13 | 09 | 11 |
| --- | --- | --- | --- | --- | --- | --- | --- | --- | --- | --- | --- | --- | --- | --- |
| 02 | 00 | 00 |  | 06 | 01 | 04 |  | 10 | 06 | 07 |  | 14 | 09 | 11 |
| 03 | 00 | 00 |  | 07 | 01 | 03 |  | 11 | 06 | 08 |  | 15 | 09 | 11 |
| 04 | 00 | 00 |  | 08 | 02 | 04 |  | 12 | 06 | 08 |  | 16 | 10 | 11 |
|  |  |  |  |  |  |  |  |  |  |  |  |  |  |  |

Application of this standard algorithm results to the pedigree gives the contributions of ancestors 01 to 04 to the other individuals. It is seen that the row for an individual is the average of the rows of its parents.

|  | 01 | 02 | 03 | 04 |
| --- | --- | --- | --- | --- |
| 01 | 1.000 | 0.000 | 0.000 | 0.000 |
| 02 | 0.000 | 1.000 | 0.000 | 0.000 |
| 03 | 0.000 | 0.000 | 1.000 | 0.000 |
| 04 | 0.000 | 0.000 | 0.000 | 1.000 |
| 05 | 0.500 | 0.000 | 0.500 | 0.000 |
| 06 | 0.500 | 0.000 | 0.000 | 0.500 |
| 07 | 0.500 | 0.000 | 0.500 | 0.000 |
| 08 | 0.000 | 0.500 | 0.000 | 0.500 |
| 09 | 0.500 | 0.000 | 0.500 | 0.000 |
| 10 | 0.500 | 0.000 | 0.250 | 0.250 |
| 11 | 0.250 | 0.250 | 0.000 | 0.500 |
| 12 | 0.250 | 0.250 | 0.000 | 0.500 |
| 13 | 0.375 | 0.125 | 0.250 | 0.250 |
| 14 | 0.375 | 0.125 | 0.250 | 0.250 |
| 15 | 0.375 | 0.125 | 0.250 | 0.250 |
| 16 | 0.375 | 0.125 | 0.125 | 0.375 |
